# Evaluation of demand and supply predictors of uptake of intermittent preventive treatment for malaria in pregnancy in Malawi

**Published:** 2017-12-01

**Authors:** Emmanuel N. Odjidja, Predrag Duric

**Affiliations:** 1 Institute of Global Health and Development, Queen Margaret University, Edinburgh, United Kingdom

## Abstract

**Background:**

The intermittent preventive treatment (IPTp) policy of Malawi (2002) stipulates that IPTp is administered during antenatal care as a direct observation therapy (DOT). The policy further recommends that IPT should be administered monthly after 16 weeks of pregnancy until delivery. This study assessed both the demand and supply factors contributing to higher dropout of IPT after the first dose. Optimal number of doses was pegged at a minimum of three in accordance with WHO recommendation.

**Materials and methods:**

Data were analysed from the Malawi multiple indicator cluster survey (2015) and the service provision assessment (2014) of 6637 women (aged 15– 49 yrs), 763 facilities and 2105 health workers. The sample was made up of pregnant women, health facilities and workers involved in routine antenatal services across all regions of Malawi. A composite indicator was constructed to report integration of IPTp with ANC services and administration of IPTp-SP as DOT. Multivariate and logistic regression were conducted to determine associations.

**Results:**

Regression analysis found that: 1. Age of women (women 35–49 yrs, AOR 1.98; 95% CI 1.42 – 2.13, number of children as well as the number of ANC visits were associated with optimal uptake of IPTp. 2. Administering IPT as DOT was higher in facilities in rural areas (AOR 1.86; 95% CI 1.54 – 1.92) than in urban areas. 3. Administration of IPTp as DOT was relatively lower in across all facilities with highest being facilities managed by CHAM (72.8%, AOR 1.40; 95% CI 1.22 – 1.54)

**Conclusion:**

Health system bottlenecks were found to present the main cause of low coverage with optimal doses of IPTp. Incorporating these results into strategic policy IPTp formulation could help improve coverage to desired levels. This study could serve as plausible evidence for government and donors when planning malaria in pregnancy interventions, especially in remote parts of Malawi.

## 1 Introduction

Malaria in pregnancy (MIP) poses an enormous public health problem that affects both the pregnant woman and her developing foetus. A recent systematic review and meta-analysis of seven trials associated MIP with severe anaemia, cerebral malaria and low neonatal birth weight (LBW) [1]. Low birthweight has been identified as the greatest risk for neonatal mortality and is among the major contributors to infant mortality [2]. Out of the 25 million pregnancies at risk of malaria globally, 85% are found in sub-Saharan Africa [3]. This results in an average of 100,000 neonatal deaths and 25,000 malaria-induced maternal deaths annually [4].

In moderate and high malaria transmission areas, WHO recommends intermittent preventive treatment (IPTp) for pregnant women during routine antenatal care (ANC) [5]. IPTp is a dose therapy of sulphadoxine-pyrimethamine (SP) administered monthly after 16 weeks of pregnancy (quickening) [5]. Available evidence suggests that three doses of SP during pregnancy are associated with fewer LBW (RR, 0.80; 95% CI, 0.69-0.94) [1] and lower incidences of cerebral malaria [6]. Among primigravidae, three doses of IPTp halves the risk of LBW and significantly reduces the possibility of preterm birth [7]. A study in Kenya found that three doses of IPTp among HIV-positive pregnant women were associated with higher prophylactic treatment against placental malaria including other pregnancy-related acute febrile illnesses [8].

Malawi, a high malaria transmission country, faces an average of 7 million malaria cases annually, which translates to 7% of all cases encountered in East- and Southern Africa [9]. Consequently, the Malawi Ministry of Health recommends a minimum of three doses of IPTp using SP which is to be administered as a DOT during antenatal care sessions [10]. While ANC attendance rate has been considerably high, uptake of the optimal doses of IPTp continue to fall below expectation [11]. According to the Malawi Demographic and Health Survey (2014), whereas 50.6% pregnant women had attended antenatal care (ANC) at least four times, only 30.0% had received a minimum of three doses of IPTp-SP. There have been contrasting findings to explain this difference. Some studies suggest that demand-related sociodemographic factors such as age, education, income, wealth and occupation are the barriers that impede optimal uptake [12–14] whilst others identify supply issues relating to periodic stock-outs, confusion over correct IPTp dosing among health workers, along with other health system bottlenecks, as key barriers [15–17].

In this study, both demand and supply factors were assessed to determine the driving determinants of low uptake in Malawi. At the demand side, multivariate analysis based on selected sociodemographic parameters were conducted whereas at the supply side, the study used indicators to measure service integration and administration of IPTp as DOT.

## 2 Materials and methods

### 2.1 Antenatal health care system in Malawi

The antenatal health care system consists of three levels of service delivery: the primary, secondary and tertiary. The primary level of care is responsible for basic diagnosis and treatment of febrile illnesses and infectious disease control whilst the secondary and tertiary levels serve as referral points for advanced care. Under the auspices of the Ministry of Health, nine technical and three supportive directorates have been tasked with the responsibility of managing the health system at the national level with the district health management teams (DHMTs) in charge of district level administration. Aside only 9% of its GDP being spent on health [18], Malawi’s healthcare is donor dependent as 68% of its health funding comes from international donors. This amounts to per capita government expenditure of US$39, which falls below the targets of the Abuja Declaration [20].

### 2.2 Study population and facilities

Secondary analysis of the Malawi Service Provision Assessment (MSPA) [20] and the multiple indicator cluster survey (MICS) [21] were conducted to determine the predictors of uptake at the demand and supply side. Both surveys were nationally representative and data were collected on key population-based socio-demographic and health indicators.

Given the focus of the study of demand predictors on IPTp uptake, 7370 women were initially selected out of the entire study population of 25,430. However, due to potential recall bias, the sample was further scaled down to include only women that reported IPTp uptake in two years preceding the survey with all doses at the antenatal care clinics. Following this criterion, a sample of 6,637 women was included in the analysis. At the supply side, analysis included two samples, which is the health facilities and health workers.

Out of the total 1060 health facilities in the Malawi service provision assessment, 763 (72.0%) were selected for this study. The inclusion criterion for selection was health facilities that offered ANC services for more than one year. Table 1 describes the distribution of selected facilities from a frame of 502 Government facilities, 190 Christian Health Association of Malawi (CHAM) facilities, 217 private and Non-Governmental (NGO) facilities and 151 facilities belonging to companies.

**Table 1:** Distribution of facilities by type and managing authority.

Facility type	Managing authority				
	Government	CHAM*	NGO	Company	Total
Hospital	48	43	2	0	93
Health centre	340	108	5	7	460
Dispensary	41	2	0	4	47
Clinic	21	9	52	58	140
Health post	22	1	0	0	23
Total	472	163	59	69	763

* Christian Health Association of Malawi

For the purpose of this study, health service workers were considered those that provided services relating to consultation, counselling, health promotion and/or laboratory services. By this definition, health workers that performed auxiliary administrative duties were excluded. The sample was statistically weighed during analysis to account for over -sampling and under-sampling of a particular cadre of staff in a facility or geographic area. This resulted in a sample of 2,105 health workers out of an original total of 4,661. The original sampling frame comprised of 59 doctors, 980 clinical technicians and medical assistants, 1,495 nurses, 35 pharmacists, 212 anaesthetists and laboratory technicians and 1,880 environmental health officers and surveillance assistants. An inclusion criterion was that health workers reported that they provided antenatal care. After applying this criterion, only 15(0.7%) specialist medical doctors, 10 (0.5%) clinical technicians, 84 (4.0%) medical assistants, 95 (4.5%) registered nurse midwife, 289 (13.7%) community health nurse, 1196 (56.8%) enrolled midwife nurse and 416 (19.8%) enrolled nurse midwife were included in the study.

### 2.3 Data and data analysis

Sociodemographic as well as economic variables that were associated with IPTp uptake were used. Wealth index, a composite economic indicator of socio-economic status was analysed as an independent variable. Similarly, other socio-demographic variables such as level of education, regional province (North, South and Central), marital status and age, were also considered as independent unit of analysis. A parity of number of children was computed.

Variables at the supply side included one main composite indicator: IPTp administered as a direct observation therapy (DOT). Administration of IPTp-SP as a DOT was an aggregation of the following variables: availability of SP at the facility, qualified and trained staff to administer SP and SP administered to a pregnant woman in the presence of a health worker.

Guided by empirical evidence [1,7] and WHO recommendations on the importance of three or more doses of IPTp during pregnancy [5] along with Malawi’s IPTp policy [22], optimal uptake was computed as a minimum of three SP doses. Low IPTp uptake was classified as fewer than this recommended number of doses. Another outcome variable considered for observation was integration of IPT services within facilities offering antenatal care services. According to the WHO [5], an effective way to ensure receipt of IPT services is via an effective integration with routine services during antenatal care. Integration was constructed as a composite of women reported to have taken the optimal dose for IPT within the antenatal care facility.

We calculated descriptive statistics of identified socio-demographic characteristics and IPT uptake. Women were classified under two groups: those who took less than 3 doses of IPT and those who received more during pregnancy. Using bivariate analysis, a test of significance was employed to assess the differences between women groups reported to have received IPTp 3+ and those who received fewer doses.

Second, a logistic regression model was employed to calculate the odds with which women with these identified characteristics will receive IPTp. The model was adjusted for facility type and cadre of health worker administering IPT. This was to ensure that all possible cofounding was controlled [14].

At the health system side, as the main outcome of study was integration of IPTp with antenatal care, we constructed a composite indicator of facilities reported to have IPTp service and situated at the same site with ANC services. Having employed multivariate analysis, a Chi-square test was used to assess differences of facilities integrating IPTp with ANC. Finally, of the facilities reported integrating IPTp with ANC, we calculated health workers in those facilities administering IPTp as DOT according to WHO recommendation [5]. This variable was disaggregated across different characteristics/cadres of health workers. Another logistic regression model was then used to assess the strength of association between health workers’ characteristics and administration of IPTp as DOT. All analyses were conducted using SPSS (version 20) and MS Excel (2013). Significance was pegged at p<0.05 (two-tailed) at 95% CI.

### 2.4 Ethical considerations

The National Health Sciences Research Committee (NHRC; Lilongwe, Malawi) and the macro institutional review board of ICF International (an international NGO in Maryland USA responsible for the demographic and health programme) and UNICEF, approved the Malawi SPA and MICS surveys, respectively. Confidentiality of data was assured during data collection and likewise, this study took intentional steps to ensure identities of respondents were not revealed at any stage of analysis. Permission for this particular study was sought from DHS MEASURE Evaluation, the MICS team and Queen Margaret University.

## 3 Results

Tables 2 and 3 show the distribution of pregnant women according to socio-economic and demographic background as well as optimal dose intake. There was a significant relationship between age group and low uptake of IPTp as 25.4% women aged 15–24 yrs received fewer than doses (AOR 1.98 95% CI 1.42–2.13) in comparison to 20.4% and 18.8% of the women aged 25–34 yrs and 35–49 yrs, respectively. In addition, women who had visited ANC a minimum of four times (which is the WHO recommended minimum number of visits) were less likely to take the recommended doses of IPTp than those who had visited less than four times (AOR 1.53 95% CI 1.29–1.82). Comparing women in the regions, those in northern Malawi (AOR 1.57 95% CI 1.37–1.72) were more likely to obtain optimal doses than their counterparts in central (AOR 1.16 95% CI 1.02–1.47) and southern Malawi (AOR 1.29 95% CI 1.21–1.38). Wealth and marital status had no significant association with three doses of IPTp. However, surprisingly, those in second quintile (AOR 1.93 95% CI 1.72–2.01) were more likely to receive IPTp 3+ in comparison to other women in other wealth groups.

**Table 2:** Distribution of pregnant women by socio-demographic characteristics and IPT uptake.

Variables	n (%) of women on IPT		P
	> 3 doses	≤ 3 doses	
Age groups (yrs)			<0.001
15–24	2002 (74.6)	681 (25.4)	
25–34	2355 (79.6)	605 (20.4)	
35–49	807 (81.2)	187 (18.8)	
Education			0.234
None	562 (77.1)	167 (22.9)	
Primary	3640 (77.4)	1063 (22.6)	
Secondary	894 (79.4)	232 (20.6)	
Higher	67 (85.9)	12 (14.1)	
Wealth quintile			0.364
Poorest	1182 (76.6)	362 (23.4)	
Second	1182 (77.3)	348 (22.7)	
Middle	1099 (77.7)	316 (22.3)	
Fourth	909 (78.8)	245 (21.2)	
Richest	792 (79.7)	202 (20.3)	
Marital status			0.286
Married	4379 (77.8)	1249 (22.2)	
Divorced/Widowed	596 (79.1)	157 (20.9)	
Never married	188 (73.7)	68 (26.3)	
Region			0.041
Northern	875 (80.5)	212 (19.5)	
Central	1799 (76.7)	548 (23.3)	
Southern	2490 (77.7)	713 (22.3)	
Number of ANC visits			<0.001
<4	2902 (80.9)	686 (19.1)	
≥4	2262 (74.2)	787 (25.8)	
Parity (# children)			<0.001
1	46 (73.0)	17 (27.0)	
2	1129 (73.9)	399 (26.1)	
≥3	2932 (79.8)	742 (20.2)	

**Table 3:** Adjusted Odds Ratios (AOR) and 95% confidence intervals (CI) of socio-demographic characteristics and uptake of IPTp (≥3 doses).

Variable	AOR	95% CI	P
Age groups (yrs)			
15–24	1.98	1.42 – 2.13	0.016
25–34	1.54	1.22 – 1.78	
35–49	1.23	1.12 – 1.36	
Education			
None	0.92	0.75 – 1.92	0.312
Primary	1.65	1.23 – 1.72	
Secondary	1.87	1.44 – 1.91	
Higher	1.92	1.77 – 2.01	
Wealth quintile			
Poorest	1.42	1.33 – 1.58	0.235
Second	1.93	1.72 – 2.01	
Middle	1.55	1.43 – 1.81	
Fourth	1.87	1.35 – 1.91	
Richest	1.39	1.12 – 1.48	
Marital status			
Married	1.92	1.71 – 2.08	0.271
Divorced/Widowed	0.93	0.72 – 1.28	
Never married	1.23	1.15 – 1.42	
Region			
Northern	1.57	1.37 – 1.72	0.022
Central	1.16	1.02 – 1.47	
Southern	1.29	1.21 – 1.38	
Number of ANC visits			
<4	1.42	1.31 – 1.59	0.018
≥4	1.53	1.29 – 1.82	
Parity (# children)			
1	1.12	1.09 – 1.32	0.020
2	1.39	1.12 – 1.49	
≥3	1.45	1.26 – 1.54	

Integration of IPTp services with antenatal care services disaggregated by selected characteristics are shown in table 4. Multivariate analysis showed that ANC facilities in northern Malawi (AOR 1.46 95% CI 1.52–1.64; Table 5) were more likely to administer IPTp during consultation compared to central and southern Malawi (P=0.041). There was a significant relationship between the managing authority and an IPTp integrated service as private facilities were less likely to administer IPTp compared to facilities managed by other entities (P<0.001). Integration of ANC and IPTp services were higher in central hospitals (100%), district hospitals (100%), rural/community hospitals (100%) and health centres (97.3%). Dispensaries, however, had the least IPT service integrated into mainstream ANC services, as 17.6% had no IPTp services at the time of the assessments. There was a significant association between facility type (p<0.001*)* and integrated IPTp services.

**Table 4:** Distribution of facilities by integration of IPT services with antenatal care services.

Integration of IPTp-SP with Antenatal Services	n (%)	95% CI	P
Region			0.041
North	115 (96.6)	92.3 – 98.7	
Central	234 (97.9)	96.2 – 98.1	
South	268 (94.0)	92.6 – 96.3	
Rural/Urban divide			0.008
Urban	107 (91.5)	90.3 – 92.7	
Rural	510 (97.0)	96.2 – 98.1	
Facility type			<0.001
Central hospital	4 (100)	-	
District hospital	24 (100)	-	
Rural/community hospital	41 (100)	-	
Other hospital	31 (83.8)	81.4 – 84.9	
Health centre	440 (97.3)	96.6 – 98.2	
Maternity	3 (75.0)	73.2 – 77.4	
Dispensary	14 (82.4)	80.0 – 84.8	
Clinic	59 (95.2)	93.5 – 97.3	
Health post	1 (50.0)	45.7 – 55.6	
Managing authority			<0.001
Government/public	396 (97.1)	96.9 – 98.1	
CHAM*	143 (97.3)	95.5 – 98.9	
Private for profit	36 (81.8)	79.2 – 83.9	
Faith-based (other than CHAM)	6 (100)	-	
NGO	9 (90.0)	89.6 – 91.7	
Company	27 (96.4)	94.5 – 98.3	

* Christian Health Association of Malawi

**Table 5:** Adjusted Odds Ratios (AOR) of health workers’ characteristics and IPTp administration as direct observation therapy (DOT) in facilities reported to have integrated IPT.

IPTp-SP administered according to guidelines (DOT)	AOR	95% CI	P
Region			0.152
North	1.46	1.52 – 1.64	
Central	1.21	1.13 – 1.38	
South	1.08	0.91 – 1.24	
Urban/Rural divide			0.004
Urban	1.29	1.12 – 1.52	
Rural	1.86	1.54 – 1.92	
Managing authority			0.012
Government	1.31	1.42 – 1.61	
CHAM*	1.40	1.22 – 1.54	
Private for profit	0.86	0.72 – 0.92	
Faith-based (other than	0.98	0.87 – 1.09	
CHAM)			
NGO	1.23	1.18 – 1.36	
Company	1.10	1.02 – 1.29	
Cadre of provider			0.038
Specialist doctor	0.23	0.13 – 0.58	
Clinical technician	0.37	0.21 – 0.64	
Medical assistant	1.38	1.19 – 1.47	
Registered midwife	1.83	1.64 – 1.96	
Registered nurse with diploma	1.52	1.23 – 1.76	
Community health nurse	1.67	1.32 – 1.92	
Enrolled midwife/Nurse Mid-	1.89	1.52 – 2.01	
wife Technical			
Enrolled nurse midwife	1.74	1.59 – 1.88	
Sex of provider			0.721
Male	1.56	1.32 – 1.78	
Female	1.41	1.29 – 1.71	

In measuring IPTp according to the recommended guidelines (i.e. as direct observation therapy) in table 6, it was found that rural facilities administered IPTp as DOT significantly more often than urban facilities (AOR 1.86 95% CI 1.54–1.92) (P=0.02). Additionally, staff in facilities managed by CHAM were more likely to administer IPTp as DOT (AOR 1.40 95% CI 1.22–1.54) than facilities managed by other entities: Government (AOR 1.31 95% CI 1.42–1.61), Private for profit (AOR 0.86 95% CI 0.72–0.92), Mission/Faith-based (other than CHAM) (AOR 0.98 95% CI 0.87–1.09), NGO (AOR 1.23 95% CI 1.18–1.36) and Company (AOR 1.10 95% CI 1.02–1.29). There was a significant association between the cadre of staff and administering IPTp-SP as a DOT (P=0.03) with the highest being registered midwives (AOR 1.89 95% CI 1.52–2.01) and specialist doctors the least (AOR 0.23 95% CI 0.13–0.58).

**Table 6:** Distribution of health staff according to administering SP as a direct observation therapy (DOT) where IPT is integrated with ANC services.

IPTp-SP administered according to guidelines (DOT)	%	95% CI	P
Region			0.163
North	74.7	70.8 – 78.3	
Central	71.0	69.3 – 71.2	
South	71.4	70.1 – 72.2	
Urban/Rural divide			0.02
Urban	61.0	59.3 – 62.3	
Rural	74.4	71.4 – 77.8	
Managing authority			0.01
Government	72.1	70.9 – 74.2	
CHAM*	72.8	70.1 – 74.9	
Private for profit	50.0	45.2 – 55.3	
Faith-based (other than CHAM)	57.1	55.1 – 59.5	
NGO	69.4	64.2 – 73.4	
Company	71.4	69.8 – 73.9	
Cadre of provider			0.03
Specialist doctor	23.3	20.5 – 26.1	
Clinical technician	35.0	31.2 – 39.2	
Medical assistant	70.8	64.1 – 75.7	
Registered midwife	77.5	73.2 – 81.2	
Registered nurse with diploma	74.3	71.2 – 77.0	
Community health nurse	70.9	68.1 – 73.1	
Enrolled midwife/Nurse Mid-wife Technical	71.3	69.2 – 74.2	
Enrolled nurse midwife	75.1	72.8 – 78.9	
Sex of provider			0.67
Male	72.8	70.6 – 74.2	
Female	71.3	68.2 – 75.1	

* Christian Health Association of Malawi

## 4 Discussion

The aim of this study was to evaluate the demand and supply predictors responsible for the low uptake of intermittent preventive treatment in Malawi. We sought to contribute to the underlying complexities which continue to affect optimal uptake of IPTp, especially in sub-Saharan Africa. To our knowledge this is the first joint analysis of SPA and MICS that assesses these factors in Malawi. Such an analytical approach provides a comprehensive understanding both from the service user and health system perspective.

Contrary to earlier studies based on the conceptual framework that IPTp uptake is influenced by sociodemographic factors (i.e. education, wealth quintile, marital status and region of residence) [12–14], we support the paradigm that optimal uptake is mainly driven by health system bottlenecks. Although findings from this study acknowledge that some parities such as the number of ANC visits and number of children were significantly associated with optimal IPTp uptake, we argue that the precursor to these barriers emanates from existing bottlenecks arising from practices of healthcare providers and the health system as a whole.

With regard to IPT administration as a DOT, we reached a conclusion that adherence to this protocol was generally lower across all facilities especially among private facilities (AOR 0.86 95% CI 0.72–0.92). Qualitative research conducted in Nigeria reinforces these findings to further suggest that weak quality standards monitoring and limited capacity to oversee services in private facilities were the main predisposition to compliance with this guideline [23]. Ouma *et al.* [24], in an ethnographic investigation, reported lack of knowledge about SP timing and confusion among health workers as barriers to successful compliance of the DOT strategy. Furthermore, Akinleye *et al.* [25] observed that although pregnant women attended ANC services and were willing to take SP doses, only 14.3% received supervision at the time of ingestion. An earlier cross-country study in Uganda, Tanzania and Malawi found that the absence of essential utilities at facilities (especially private facilities) such as cups were factors constraining successful optimal uptake. Together with our findings, we wholly support efforts to train health workers especially within private facilities in urban settings. In addition, we call attention of government actors and donors to strengthen MIP quality improvement and supportive supervision especially at the primary level of care.

Integration of IPTp services with antenatal care has been recognised as a cost-effective mechanism to increase IPTp uptake as well as provide comprehensive antenatal care [26]. While results from our study confirm that an average of 67.7% of facilities integrated IPTp services with antenatal care, facilities managed by private entities and CHAM fell below this average. Integration of control of malaria in pregnancy services with antenatal care improves relationships between pregnant women and provider which eventually increases ANC attendance [26]. A systematic review on socio-cultural factors affecting uptake of MIP interventions in Africa confirmed this finding [17]. According to authors, a dominant factor that encouraged discontinuity of subsequent SP doses after the initial one was travelling to fragmented points of care for malaria in pregnancy services. Another meta-analysis reinforced these findings to illustrate how intermittent stock of SP in health centres had resulted to seeking care in higher level facilities [15]. The authors contend that inadequate transportation means together with high cost of travelling had given rise to higher dropout rates of IPTp especially in hard to reach communities. Two cross-sectional studies funded by WHO in Tanzania partially attributed these to lack of real-time health information system that allows health managers to respond as well as a poor road network [27]. To remedy this, we encourage health investments to be channelled to strengthening the district health information system (DHIS) to capture and disseminate real-time information on drug availability especially in hard to reach areas.

Our study had some limitations. First, the nature of this study being cross-sectional limits us from claiming causality. At the demand side, the study only included respondents with live births two years preceding the study. Therefore, women that experienced perinatal death or still born were excluded from the study. Another source of potential bias was during data collection of the pregnant women. Since data on IPTp uptake was self-reported, it could have resulted to recall bias. In this instance, recall bias could result in underestimating or overestimating the magnitude of IPTp uptake. Finally, other factors were not explored as we were limited on the variables available in both data sets. At the demand side, for instance, variables such as beliefs and perceptions of pregnant women and their spouses on IPTp-SP would have been useful in understanding context-specific issues that affects optimisation. In the context of these limitations, we recommend that future research should focus on an ethnographic research to explore how women, their spouses and the community as a whole understand malaria during pregnancy and uptake of MIP interventions.

## 5 Conclusions

The lacuna in ANC coverage and IPTp uptake underscores missed opportunities at antenatal care in Malawi. This study presents another paradigm to understanding the complexity around low uptake in Malawi which resonates among other sub-Sahara African countries. Three themes emanated from this study: limited implementation of IPTp as DOT, low uptake which driven by identified health system bottlenecks notably low integration of IPTp with ANC services and higher dropouts after first due to limited service integration in facilities.

While integration of IPTp with antenatal care has the potential to encourage future uptake, its successful achievement would depend on a strengthened health system that responds to stock outs in a timely manner. This implies that commitment to health system strengthening efforts by government and donors will be critical. Finally, given the relevance of administering IPTp as a DOT to monitoring and surveillance, health authorities should continually seek to reinforce this through supportive quality improvement and providing MIP guidelines in local languages as well as promoting efforts to improve service integration on the ANC platform.

## References

[1] Kayentao K, Garner P, Van Eijk AM, Naidoo I (2013). Intermittent preventive therapy for malaria during pregnancy using 2 vs 3 or more doses of sulfadoxine-pyrimethamine and risk of low birth weight in Africa.. JAMA.

[2] Guyatt H, Snow R (2004). Impact of malaria during pregnancy on low birth weight in sub-Saharan Africa.. Clin. Microbiol. Rev..

[3] Dellicour S, Tatem A, Guerra C, Snow R (2010). Quantifying the number of pregnancies at risk of malaria in 2007: A demographic study.. PLoS Med..

[4] Malaria in Pregnancy Consortium. MiP - Malaria in Pregnancy Information on Maternal and neonatal deaths.. http.//www.mip-consortium.org/about_us/mission.htm.

[5] World Health Organisation. (2014). WHO Policy brief for the implementation of intermittent preventive treatment of malaria in pregnancy using Sulfadoxine-Pyrimethamine (IPTp-SP)..

[6] Okolie V, Obiechina NJ, Okechukwu ZC, Oguejiofor CF (2014). Placental malaria and its associations with pregnancy outcome.. Int. J. Trop. Dis. Health.

[7] Diakite OS, Kayentao K, Traoré BT, Djimde A (2011). Superiority of 3 over 2 doses of intermittent preventive treatment with sulfadoxine-pyrimethamine for the prevention of malaria during pregnancy in Mali: A randomized controlled trial.. Clin. Infect. Dis..

[8] Van Eijk AM, Ayisi JG, ter Kuile FO, Slutsker L (2004). Effectiveness of intermittent preventive treatment with sulphadoxine-pyrimethamine for control of malaria in pregnancy in western Kenya: a hospital-based study.. Trop. Med. Int. Health.

[9] World Health Organization. (2016). World Malaria Report 2016..

[10] Feng G, Simpson JA, Chaluluka E, Molyneux ME (2010). Decreasing burden of malaria in pregnancy in Malawian women and its relationship to use of intermittent preventive therapy or bed nets.. PLoS One.

[11] ICF International (2017). The DHS Program - Data.. DHS program..

[12] Kibusi SM, Kimunai E, Hines CS (2015). Predictors for uptake of intermittent preventive treatment of malaria in pregnancy (IPTp) in Tanzania.. BMC Public Health.

[13] Van Eijk AM, Hill J, Larsen DA, Webster J (2013). Coverage of intermittent preventive treatment and insecticide-treated nets for the control of malaria during pregnancy in sub-Saharan Africa: a synthesis and meta-analysis of national survey data, 2009–11.. Lancet Infect. Dis..

[14] Chepkemoi Ng’etich Mutulei A. (2013). Factors influencing the uptake of intermittent preventive treatment for malaria in pregnancy: Evidence from Bungoma East District, Kenya.. Am. J. Publ. Health Res..

[15] Hill J, Kazembe P (2006). Reaching the Abuja target for intermittent preventive treatment of malaria in pregnancy in African women: a review of progress and operational challenges.. Trop. Med. Int. Health.

[16] Launiala A, Honkasalo ML (2007). Ethnographic study of factors influencing compliance to intermittent preventive treatment of malaria during pregnancy among Yao women in rural Malawi.. Trans. R. Soc. Trop. Med. Hyg..

[17] Pell C, Straus L, Andrew E, Meñaca A (2011). Social and cultural factors affecting uptake of interventions for malaria in pregnancy in Africa: A systematic review of the qualitative research.. PLoS One.

[18] World Bank. Malawi Data.. http://data.worldbank.org/country/malawi.

[19] Bowie C (2006). The burden of disease in Malawi.. Malawi Med. J..

[20] Ministry of Health, Malawi and ICF International. (2014). Malawi Service Provision Assessment (MSPA) 2013-14..

[21] UNICEF. Surveys-UNICEF MICS 2017.. http://mics.unicef.org/surveys.

[22] Mwendera CA, De Jager C, Longwe H, Phiri K (2017). Changing the policy for intermittent preventive treatment with sulfadoxine–pyrimethamine during pregnancy in Malawi.. Malar. J..

[23] Ameh S, Owoaje E, Oyo-Ita A (2016). Barriers to and determinants of the use of intermittent preventive treatment of malaria in pregnancy in Cross River State, Nigeria: a cross-sectional study.. BMC Pregnancy Childbirth.

[24] Ouma P, Van Eijk A, Hamel M (2007). The effect of health care worker training on the use of intermittent preventive treatment for malaria in pregnancy in rural western Kenya.. Trop. Med. Int. Health.

[25] Akinleye S, Falade C, Ajayi I (2009). Knowledge and utilization of intermittent preventive treatment for malaria among pregnant women attending antenatal clinics in primary health care centers in rural southwest, Nigeria: a cross-sectional study.. BMC Pregnancy Childbirth.

[26] De Jongh TE, Gurol–Urganci I, Allen E, Zhu NJ Integration of antenatal care services with health programmes in low–and middle–income countries: systematic review.. J. Glob. Health,.

[27] Mubyazi G, Bloch P, Kamugisha M, Kitua A (2005). Intermittent preventive treatment of malaria during pregnancy: a qualitative study of knowledge, attitudes and practices of district health managers, antenatal care staff and pregnant women in Korogwe District, North-Eastern Tanzania.. Malar. J..

